# AI-driven segmentation of the pulp cavity system in mandibular molars on CBCT images using convolutional neural networks

**DOI:** 10.1007/s00784-024-06009-2

**Published:** 2024-11-21

**Authors:** Marie Louise Slim, Reinhilde Jacobs, Renata Maíra de Souza Leal, Rocharles Cavalcante Fontenele

**Affiliations:** 1 OMFS-IMPATH Research Group, Department of Imaging and Pathology, Faculty of Medicine, KU Leuven, Kapucijnenvoer 7, Leuven, 3000 Belgium; 2 Department of Endodontics, Faculty of Dentistry, Saint Joseph University, Beirut, Lebanon; 3 Department of Dental Medicine, Karolinska Institute, Stockholm, Sweden; 4 Department of Restorative Dentistry, Federal University of Paraná, Curitiba, Paraná Brazil

**Keywords:** Artificial intelligence, Convolutional neural network, Dental pulp, Cone-beam computed tomography, Mandibular molars

## Abstract

**Objective:**

To develop and validate an artificial intelligence (AI)-driven tool for automated segmentation of the pulp cavity system of mandibular molars on cone-beam computed tomography (CBCT) images.

**Materials and methods:**

After ethical approval, 66 CBCT scans were retrieved from a hospital database and divided into training (*n* = 26, 86 molars), validation (*n* = 7, 20 molars), and testing (*n* = 33, 60 molars) sets. After automated segmentation, an expert evaluated the quality of the AI-driven segmentations. The expert then refined any under- or over-segmentation to produce refined-AI (R-AI) segmentations. The AI and R-AI 3D models were compared to assess the accuracy. 30% of the testing sample was randomly selected to assess accuracy metrics and conduct time analysis.

**Results:**

The AI-driven tool achieved high accuracy, with a Dice similarity coefficient (DSC) of 88% ± 7% for first molars and 90% ± 6% for second molars (*p* > .05). The 95% Hausdorff distance (HD) was lower for AI-driven segmentation (0.13 ± 0.07) compared to manual segmentation (0.21 ± 0.08) (*p* < .05). Regarding time efficiency, AI-driven (4.3 ± 2 s) and R-AI segmentation (139 ± 93 s) methods were the fastest, compared to manual segmentation (2349 ± 444 s) (*p* < .05).

**Conclusion:**

The AI-driven segmentation proved to be accurate and time-efficient in segmenting the pulp cavity system in mandibular molars.

**Clinical Relevance:**

Automated segmentation of the pulp cavity system may result in a fast and accurate 3D model, facilitating minimal-invasive endodontics and leading to higher efficiency of the endodontic workflow, enabling anticipation of complications.

## Introduction

Every year, 23 million root canal treatments are performed in the European Union [1]. The complexity inherent in each tooth’s root canal system poses a significant challenge in terms of shaping, cleaning, and disinfecting these structures [2]. Addressing this challenge requires robust imaging techniques to accurately visualize the internal anatomy of the tooth and guide treatment strategies [2].

Radiographic imaging is an essential tool in endodontics, providing the foundational data necessary for accurate diagnosis and effective treatment planning [3]. Cone-beam computed tomography (CBCT) offers three-dimensional (3D) imaging of the area under examination through a series of multiplanar reconstructions (i.e., axial, coronal, and sagittal views) [4]. The 3D imaging provided by CBCT is especially valuable for endodontics, allowing clinicians to gain a better understanding of dentomaxillofacial anatomical structures, which in turn improves the detection of endodontic diseases and facilitates more effective treatment planning [5, 6]. As techniques continue to evolve, CBCT can be integrated with sophisticated modelling software to generate 3D models. These models play a pivotal role in improving endodontic diagnosis and guiding treatment, allowing clinicians to visualize internal tooth anatomy with greater accuracy. This level of detail is valuable in complex endodontic cases, where traditional imaging might not offer sufficient information, thus enabling more precise treatment planning and leading to improved patient outcomes [7].

Segmentation is a fundamental technique in medical image analysis that involves identifying and isolating specific regions of interest within an image. This technique has initiated a new phase of virtual treatment planning, which leverages 3D surface models derived from segmented Digital Imaging and Communications in Medicine (DICOM) data. These 3D models are integrated into virtual treatment planning software, allowing clinicians to visualize the 3D anatomy and simulate various procedures. This innovative approach to treatment planning enables more refined and optimized therapeutic strategies, leading to enhanced precision and improved patient outcomes [8]. Traditional segmentation methods can be challenging due to issues such as partial volume effect, artefacts resulting from high-density materials such as metal objects and radiopaque materials like endodontic sealers, and poor image quality. Additionally, these methods are susceptible to human subjectivity, and are often time-consuming [9–11].

Convolutional Neural Networks (CNNs), which are robust deep learning algorithms, have improved the segmentation of dentomaxillofacial structures on CBCT scans, overcoming the limitations of traditional methods [12, 13]. Segmentation plays a pivotal role in endodontics by enabling precise identification and isolation of specific anatomical structures, which is critical for accurate diagnosis, personalized treatment planning, and improved patient outcomes. Through segmentation, endodontists can clearly visualize pulp anatomy, identify apical foramina, and detect anatomical variations. This precision allows for tailored treatment plans, especially in complex cases with varying root canal shapes and lengths. It aids minimal invasive endodontic therapy, facilitating finding access to different pulp horns, even in case of pulpal obliteration. Furthermore, segmentation also facilitates guided endodontics, using 3D-printed guides to ensure minimal invasiveness by precise access to root canals and a reduced risk of unnecessary dentin removal [14]. It is essential for post-treatment assessment, allowing for effective monitoring of treatment success and early detection of complications. Furthermore, segmentation may enhance communication amongst dental professionals and patients, while serving as a valuable tool for training, research and innovation, which might aid advancements in endodontic technologies and therapies for enhanced endodontic quality care [15, 16].

While segmentation of the tooth, root, and pulp cavity system has been used for various endodontic tasks, segmenting the pulp cavity system in mandibular molars is particularly complex and time-consuming, making it uncommon in clinical practice [17]. The introduction of AI for molar pulp cavity system segmentation has the potential to overcome this burden in an accurate and time-efficient approach. Therefore, the objective of this study was to develop and validate an AI-driven tool for segmentation of the pulp cavity system in mandibular molars using CBCT scans. The null hypothesis was that the AI-driven tool would not be able to provide an accurate and time-efficient segmentation of the pulp cavity system in mandibular molars.

## Materials and methods

### Ethical criteria

This retrospective study was conducted following local ethics committee approval (protocol number S67798) and in compliance with the World Medical Association Declaration of Helsinki on medical research. All patient data were anonymized. The present study followed the Checklist for Artificial Intelligence in Medical Imaging (CLAIM) guidelines [18].

### CBCT data collection

A total of 66 CBCT scans were retrospectively collected from the radiology database of the UZ Leuven Hospital, Leuven, Belgium. The CBCT scans were acquired with two devices, the NewTom VGi evo (QR Verona, Cefla, Verona, Italy) and the 3D Accuitomo 170 (Morita, Kyoto, Japan), each utilizing different acquisition protocols to create a heterogeneous dataset (Table 1).

**Table 1:** Cone-beam computed tomography dataset acquisition parameters

CBCT device	kVp	mA	FOV	Voxel size (mm^3^)
3D Accuitomo 170	90	5	6 × 6, 8 × 8, 10 × 10, 14 × 10,17 × 12 cm	0.125–0.250
NewTom VGI evo	110	3–17	8 × 8, 10 × 10, 12 × 8, 15 × 15, 24 × 19 cm	0.125-0.300

*Abbreviations*: CBCT, cone-beam computed tomography; kVp, kilovoltage peak; mA, tube current; FOV, field of view; mm, millimeters

Inclusion criteria consisted of CBCT scans with acceptable subjective quality, demonstrating adequate sharpness and contrast to ensure clear delineation of pulp chambers and root canals. Additionally, CBCT scans needed to have a field of view (FOV) that included the mandibular teeth. Scans with significant artefacts from high-density materials, such as multiple dental implants in the same arch, or with significant motion artefacts, were excluded.

**Fig. 1:**
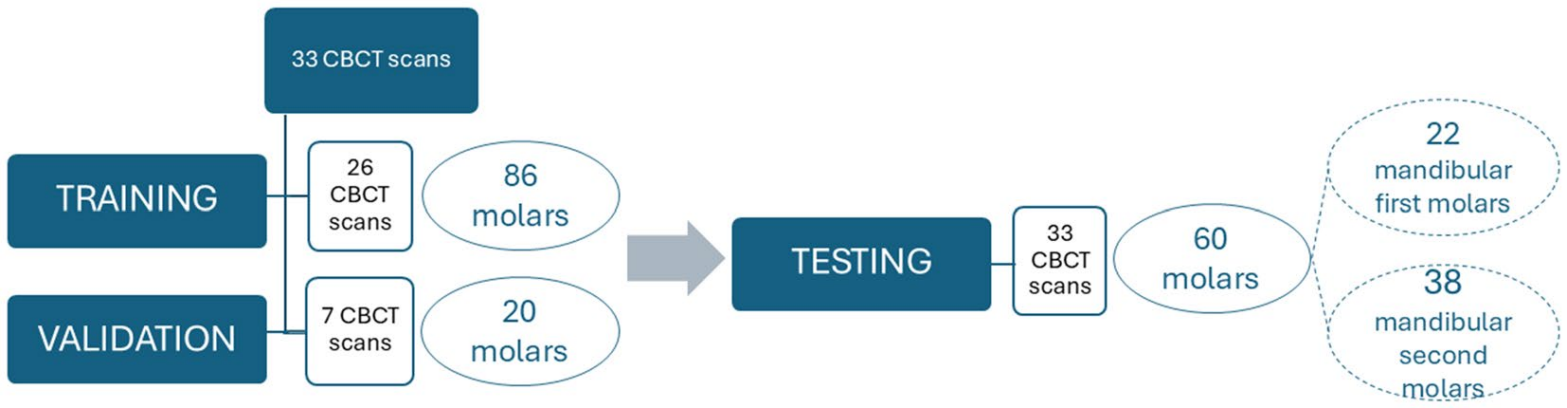
Flowchart of the cone-beam computed tomography dataset used for training, validation, and testing of the convolutional neural network model

Selected CBCT scans were randomly divided into three subsets. The first subset consisted of 26 CBCT scans (86 molars) and was used to train the CNN model through manual segmentation of the pulp cavity system. A second subset of 7 CBCT scans (20 molars) was used as a validation set to determine the optimal model architecture and to avoid overfitting the CNN model. The third subset of 33 CBCT scans (60 molars) was used as a testing set to evaluate the performance of the AI-driven tool by comparing the automatic segmentation of the CNN model with the expert refined AI (R-AI) segmentation. The flowchart in Fig. 1 provided an overview of the dataset. All CBCT scans were exported in DICOM format.

### Ground-truth labelling

CBCT scans were uploaded to Virtual Patient Creator (Relu, Leuven, Belgium). This tool is designed for automated 3D segmentation of CBCT-derived dentomaxillofacial structures. While the software is primarily automated, it also allows for manual delineation of regions of interest. Users can utilize features such as the brush, contour, and interpolation tools to perform slice-by-slice segmentation across three orthogonal planes (axial, coronal, and sagittal) using a voxel-wise approach. To create the ground-truth datasets for training and validating the CNN model, the pulp cavity system of each mandibular molar in the CBCT training and validation datasets was manually delineated using the tools available in the Virtual Patient Creator platform. This task was performed by two experienced operators, one endodontist and one oral radiologist (MLS and RCF), both with over five years of experience. Once the manual segmentations were completed, a 3D model of the pulp chamber and root canals was exported in Standard Tessellation Language (STL) file format for further processing. Before proceeding with AI training, both observers reviewed all segmentations during a consensus meeting to identify any potential flaws. If necessary, further adjustments were made using the brush tool to accurately delineate the region of interest in the axial, coronal, and sagittal planes.

### CNN model architecture

The AI segmentation pipeline involved two sequential 3D U-Net neural networks, each comprising four encoder blocks and three decoder blocks. Each block contained two convolutions with a 3 × 3 × 3 kernel, followed by a rectified linear unit (ReLU) activation, group normalization with eight feature maps, and max pooling operations that reduced the spatial resolution by a factor of 2 in all dimensions. The design of the U-Net architecture allowed for effective segmentation of dentomaxillofacial structures in CBCT scans, particularly with large FOV. A two-step method was employed, as recommended by previous studies [10, 17]. In the first step, the initial U-Net model produced a preliminary segmentation of the region of interest. The second U-Net model then refined this segmentation to achieve full-resolution accuracy for the final segmentation of pulp cavity system.

The CNN models were implemented using PyTorch, with data augmentation techniques such as random cropping, scaling, rotation, mirroring, and elastic deformation to enhance the training dataset and improve the robustness of the model. Additionally, the ADAM optimization algorithm was applied to optimize model parameters, starting with a learning rate of 1.25e^− 4^, which was halved seven times over 300 epochs. Early stopping was utilized based on the validation set to prevent overfitting by indicating a saturation point where additional training data would lead to data overfitting without any improvements in the model performance. The CNN model was then internally validated and implemented on the cloud-based platform Virtual Patient Creator.

### Testing step - Automated segmentation of the pulp cavity system

**Fig. 2:**
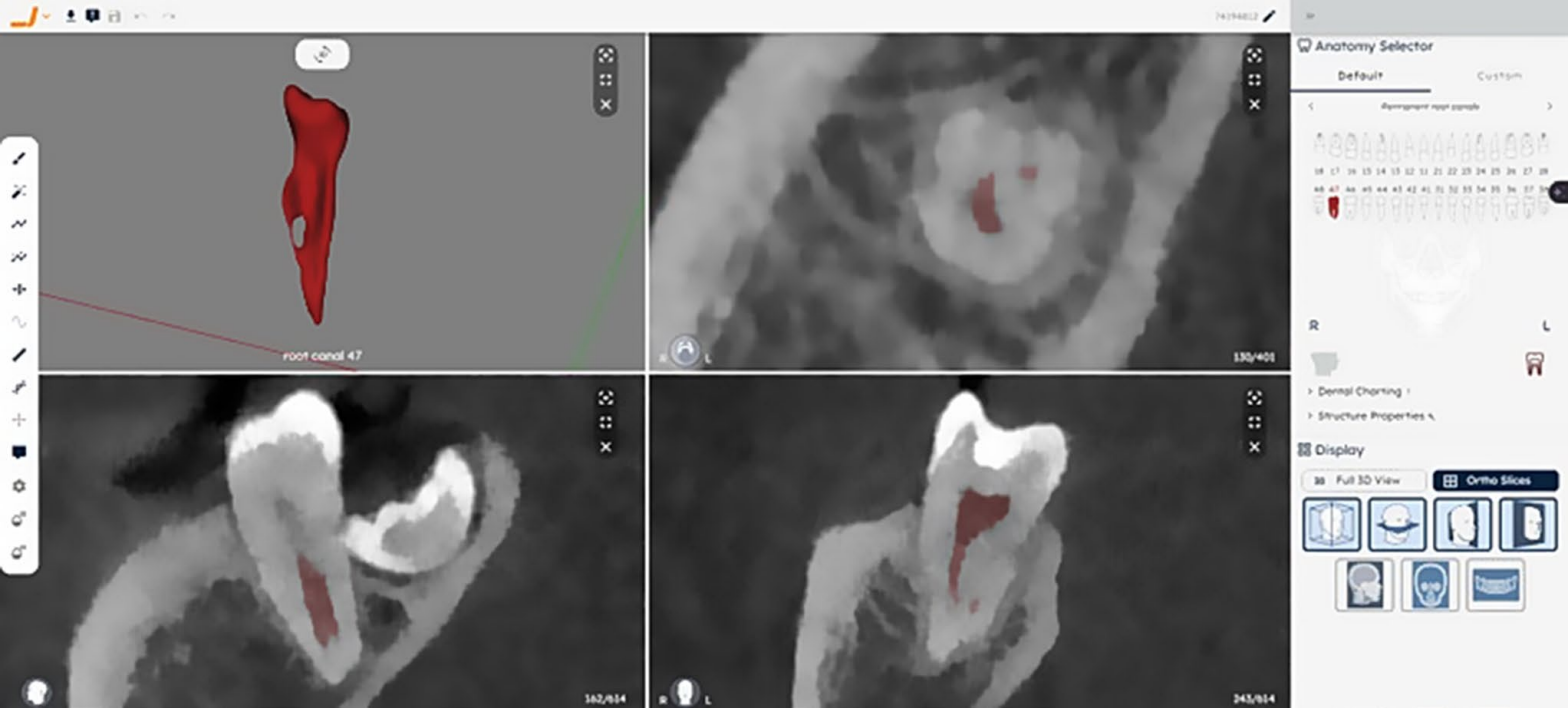
Three-dimensional model of the pulp cavity system segmentation of a mandibular right second molar on axial, coronal and sagittal reconstructions in the “Virtual Patient Cfreator” (Relu, Leuven, Belgium)

Each CBCT scan, in DICOM format, was uploaded to the Virtual Patient Creator platform. This platform used a multi-class approach to automatically segment pulp cavity system. After segmentation, it generated individual 3D models of the pulp chamber and root canals for each tooth, which were exported in STL format. The time required for automatic segmentation of the pulp was recorded with a digital stopwatch (Fig. 2).

### Testing step - Refinement of the automated segmentation of pulp cavity system

**Fig. 3:**
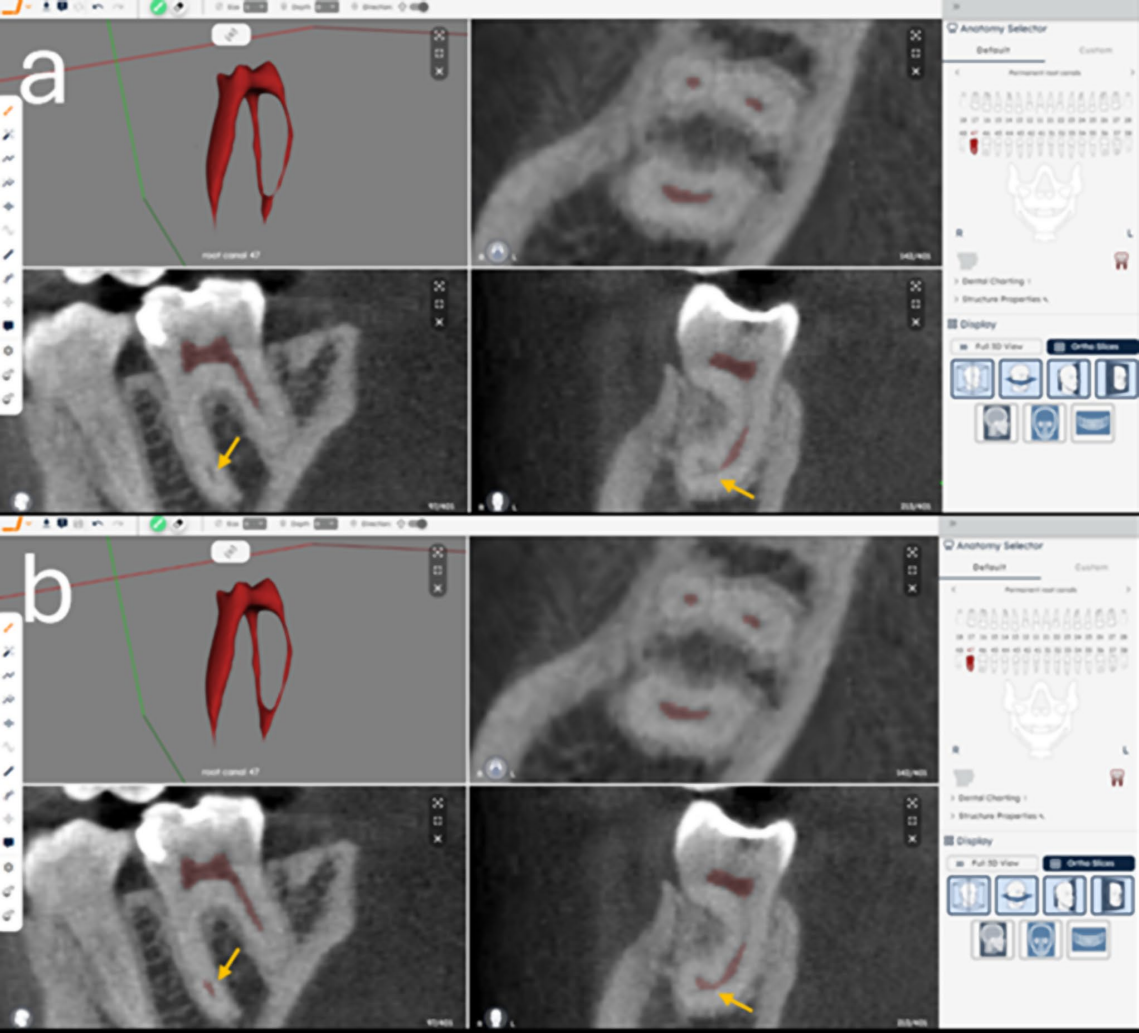
Three-dimensional model of the pulp cavity system of a mandibular right second molar and its integrated automated segmentation (**a**) and refined segmentation (**b**) on axial, coronal and sagittal reconstructions in the “Virtual Patient Creator” (Relu, Leuven, Belgium)

One operator (MLS) reviewed and refined the AI-driven automated segmentations to correct potential errors in the AI segmentation, which could include under- and/or over-segmentation. This process was conducted on the cloud-based platform Virtual Patient Creator. The CBCT scans carefully examined using axial, sagittal, and coronal multiplanar reconstructions to identify any discrepancies. Identified segmentation errors were corrected using the brush tool, which allowed the operator to add or remove voxels in the segmentation maps. To enhance the visualization of the internal anatomy of each mandibular molar and facilitate comparison with the AI segmentation map contours, the resliceable axes tool was used. This tool allows for adjustments to the axial, sagittal, and coronal planes, aligning them to be parallel and perpendicular relative to the region of interest (Fig. 3). After the refinements were completed, the refined-AI (R-AI) 3D models were exported in STL format. The time required for manual refinement was recorded using a digital stopwatch.

### Accuracy

The accuracy metrics used to assess the performance of the AI-driven tool were calculated based on a comparison of the AI and R-AI 3D models using a confusion matrix at the voxel level for a binary segmentation task. From this analysis, the following variables were quantified: true positive (TP), false positive (FP), true negative (TN), and false negative (FN) [19–21].

1. **TP**: Voxels that were correctly identified as part of the pulp cavity system in the automated segmentation.
2. **FP**: Voxels that were erroneously included in the automated segmentation and subsequently removed during pulp cavity system refinement.
3. **TN**: Voxels that did not represent pulp cavity system and were accurately excluded from the final segmentation map.
4. **FN**: Voxels that were not included in the automated segmentation but were later added during the refinement process.

Based on the aforementioned variables, the following accuracy metrics were measured for assessing the performance of the AI-driven segmentation (Table 2).

**Table 2:** Accuracy metrics of the AI-driven tool for automated pulp cavity system segmentation of mandibular molars

	Accuracy metrics					
	IoU (%)	DSC (%)	Recall (%)	Precision (%)	Accuracy (%)	95% HD (mm)
1st versus 2nd molar (n)	Mean (SD)					
1st molar (22)	80 (12)	88 (7)	86 (9)	91 (6)	99 (1)	0.14 (0.10)
2nd molar (38)	82 (10)	90 (6)	88 (7)	93 (4)	99 (1)	0.15 (0.09)
*p*-value	0.41	0.30	0.46	0.26	0.34	0.84

*Abbreviations*: IoU, Intersection over union; DSC, Dice similarity coefficient; HD, Hausdorff distance; n, number of teeth; SD, Standard deviation

- **Intersection over Union (IoU)**: Measures the degree of overlap between the AI segmentation map and the R-AI segmentation map.
  $$\varvec{IoU}(\varvec{A},\varvec{B})=\:\frac{|\:\text{A}\cap\:\text{B}|\:\:}{\:|\:\text{A}\cup\:\text{B}\:|\:}=\frac{\text{T}\text{P}}{\text{T}\text{P}\:+\:\text{F}\text{P}\:+\:\text{F}\text{N}}$$
- **Dice Similarity Coefficient (DSC)**: Assesses the degree of intersection between the AI segmentation map and the R-AI segmentation map.
  $$\:\varvec{D}\varvec{S}\varvec{C}\:\left(\mathbf{A},\:\mathbf{B}\right)=\:\frac{2\:\:|\mathbf{A}\cap\:\mathbf{B}|\:}{\:\left|\mathbf{A}\right|+\:\left|\mathbf{B}\right|}=\frac{2\:\times\:\mathbf{T}\mathbf{P}}{2\times\:\mathbf{T}\mathbf{P}+\mathbf{F}\mathbf{P}+\mathbf{F}\mathbf{N}}$$
- **Recall**: Indicates the ratio of voxels that were truly part of the pulp cavity system and were accurately detected by the AI tool.
  $$\varvec{Recall}=\frac{\text{T}\text{P}}{\text{T}\text{P}\:+\:\text{F}\text{N}}$$
- **Precision**: Represents the ratio of correctly identified voxels to the total number of voxels that the AI tool classified as part of the pulp.
  $$\varvec{Precision}\frac{\text{T}\text{P}}{\text{T}\text{P}\:+\:\text{F}\text{P}}$$
- **Accuracy**: Denotes the ratio of voxels accurately detected among all observed voxels.
  $$\varvec{Accuracy}=\frac{\text{T}\text{P}\:+\:\text{T}\text{N}}{\text{T}\text{P}\:+\:\text{T}\text{N}\:+\:\text{F}\text{P}\:+\:\text{F}\text{N}}$$
- **95% Hausdorff Distance (HD)**: Represents the 95th percentile of the maximum distance between any point on the AI segmentation map and the closest corresponding point on the R-AI segmentation map. Using the 95th percentile helps mitigate the impact of outliers. If the 95% HD is 0 millimeters (mm) and the other metrics are at 100%, this represents a perfect segmentation.
  $$95\%\varvec{HD}=\begin{array}{ll}{\text{P}95(\min\Vert \text{p}-\text{g}}&{\Vert 2\text{u}\min\Vert\text{g}-\text{p}\Vert 2)}\\{{\text{g}\in \text{G}}}&{{\text{p}\in \text{P}}}\end{array}$$

### Analysis of the required segmentation time

To determine the average time needed to create segmentation maps, AI, R-AI, and manual methods were investigated.

1. **Manual Method**: Two experts (MLS and RMSL) conducted manual segmentation, with the time recorded using a digital stopwatch from the import of the CBCT data to the creation of the final segmentation map. Each expert performed the segmentation twice, with a 30-day interval between sessions, to evaluate intra-operator reliability. This reliability was assessed using metrics including the 95% HD, IoU, precision, recall, and accuracy.
2. **AI Method**: The time required for obtaining the final segmentation map on the online platform was recorded using a digital stopwatch.
3. **R-AI Method**: The time for manual refinements was added to the AI method’s segmentation time, providing the combined time for the R-AI method.

### Statistical analysis

Statistical analysis was performed using SPSS software (version 24.0, IBM Corp., Armonk, NY). Descriptive analysis summarized the accuracy metrics and timing data in this study, presented as averages and standard deviations. Accuracy metrics, including IoU, DSC, Recall, Precision, Accuracy, and the 95% HD were calculated. Independent t-test was used to compare each accuracy metric between the first and second mandibular molars. Additionally, paired t-test was utilized to evaluate the differences in performance between manual and AI segmentation for each accuracy metric. To compare the time taken to generate 3D root canal models across the different segmentation methods (manual, AI, and R-AI), one-way Analysis of Variance (ANOVA) was used, followed by a Tukey post-hoc test. The significance level was set at 5% for all analyses (*p* ≤ .05).

## Results

**Fig. 4:**
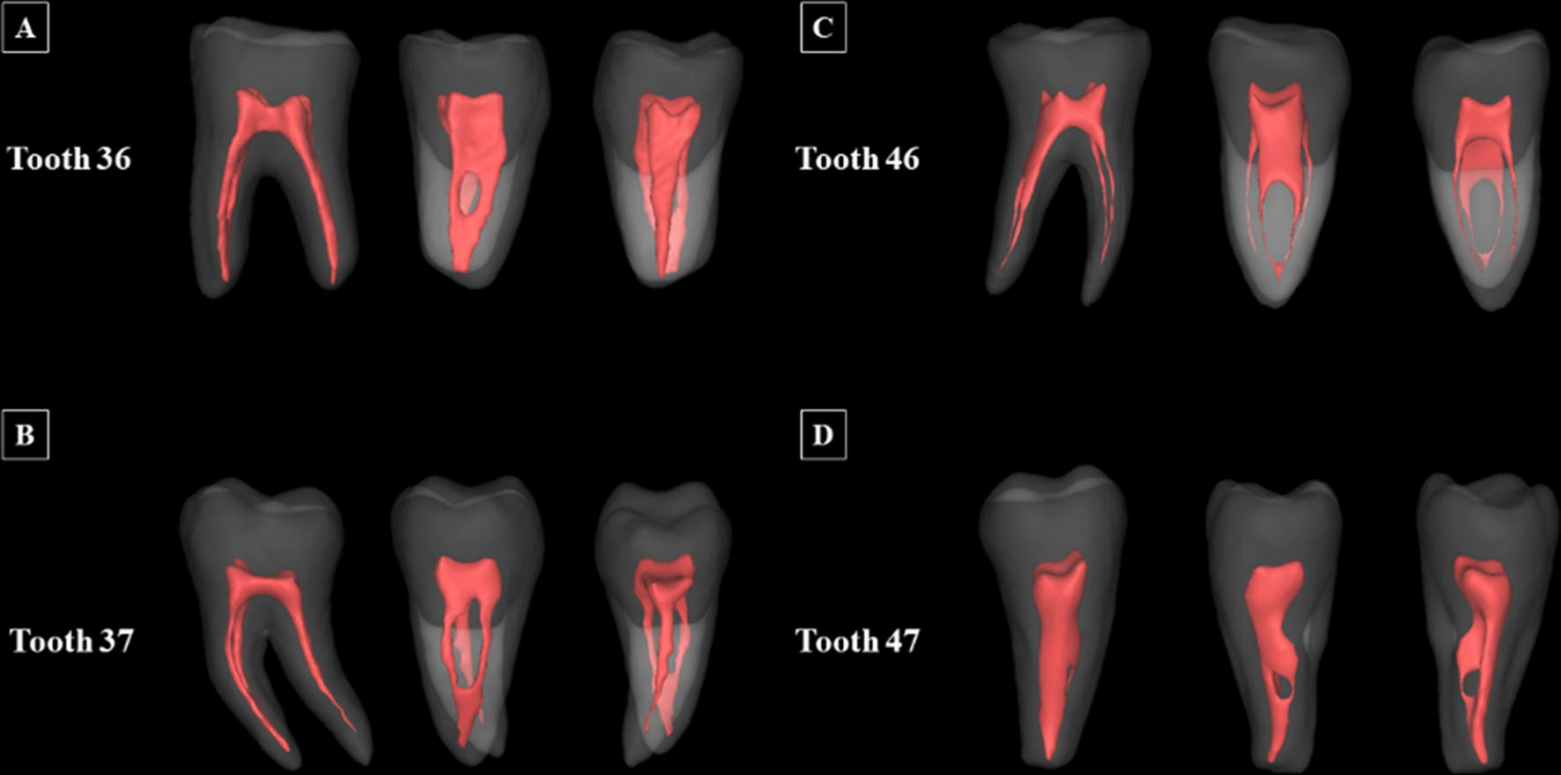
Three-dimensional models of mandibular molars and their integrated pulp cavity system segmentation

Out of 60 segmented mandibular molars, 11 molars required no refinement after automated segmentation. Figure 4 illustrates the high performance of the AI-driven tool by depicting 3D model of mandibular molars with the segmented pulp cavity system overlaid. Table 2 summarizes the overall performance of the AI-driven tool in generating 3D models of the pulp cavity system models for mandibular molars, based on the accuracy metrics.

Accuracy metrics were not significantly different for first and second mandibular molars (*p* > .05). The results indicated high performance, with IoU values of 80% ± 12 for the mandibular first molars and 82% ± 10 for the mandibular second molars, DSC of 88% ± 7 for the mandibular first molars and 90% ± 6 for the mandibular second molars, Recall of 86% ± 9 for the mandibular first molars and 88% ± 7 for the mandibular second molars, Precision of 91% ± 6 for the mandibular first molars and 93% ± 4 for the mandibular second molars, and overall Accuracy of 99% ± 1% for both mandibular first and second molars. Moreover, the 95% HD demonstrated low values of 0.14 ± 0.1 mm for the mandibular first molars and 0.15 ± 0.09 mm for the mandibular second molars. These outcomes indicate that the AI-driven tool achieved accurate automatic segmentation of pulp cavity system, closely aligning with the R-AI segmentation, suggesting only minor refinements were necessary.

**Fig. 5:**
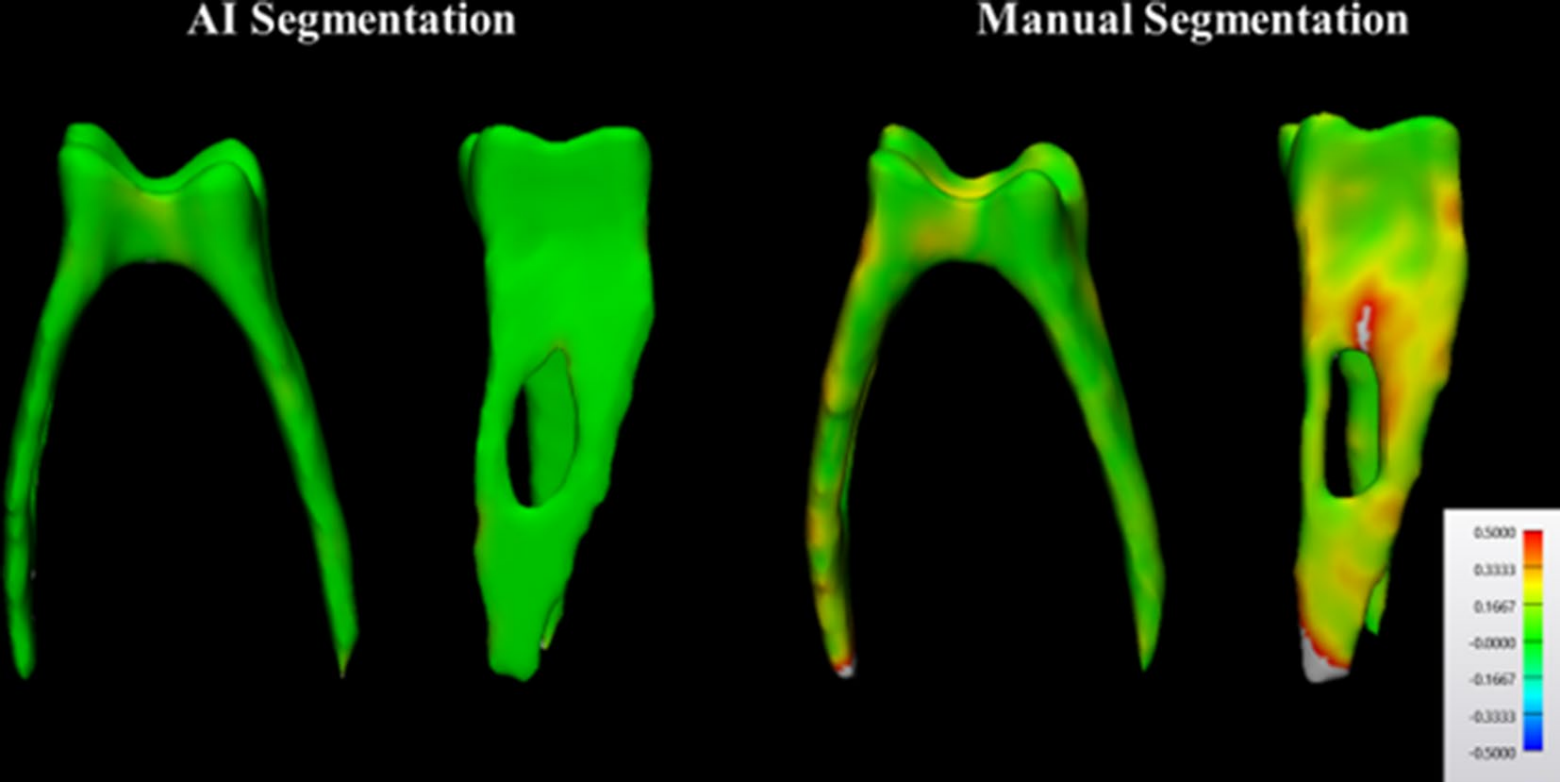
Comparison of artificial intelligence-driven and manual three-dimensional models for a mandibular left first molar employing color mapping in frontal and lateral views

Table 3 presents the results regarding the comparison between manual and AI segmentation approaches in terms of accuracy. The results for IoU, DSC, precision, and accuracy were similar for both AI and manual methods (*p* > .05). However, the AI segmentation method outperformed the manual method in recall and 95% HD values (*p* < .05). Figure 5 highlights the higher accuracy of the AI segmentation map, where most of the tooth is depicted in green, indicating a high level of alignment with the R-AI 3D model. In contrast, the manual segmentation shows a 3D model with more discrepancies, highlighted in yellow and red.

**Table 3:** Comparison between human (manual) and AI segmentations in terms of mean ± standard deviation for accuracy metrics

Metric	Human (Manual segmentation)	AI	*p*-value
IoU (%)	77 ± 9.3	83 ± 11	0.09
DSC (%)	86.4 ± 5.8	91 ± 6.3	0.08
Recall (%) *	84 ± 8	90 ± 8.2	0.05
Precision (%)	90.3 ± 7.6	92 ± 4.5	0.52
Accuracy (%)	98.3 ± 0.8	99 ± 0.8	0.09
95% HD (mm) *	0.21 ± 0.08	0.13 ± 0.07	0.009

Asterisks (*) indicate statistically significant differences between human and AI performance within each accuracy metric (*p* ≤ .05)

**Fig. 6:**
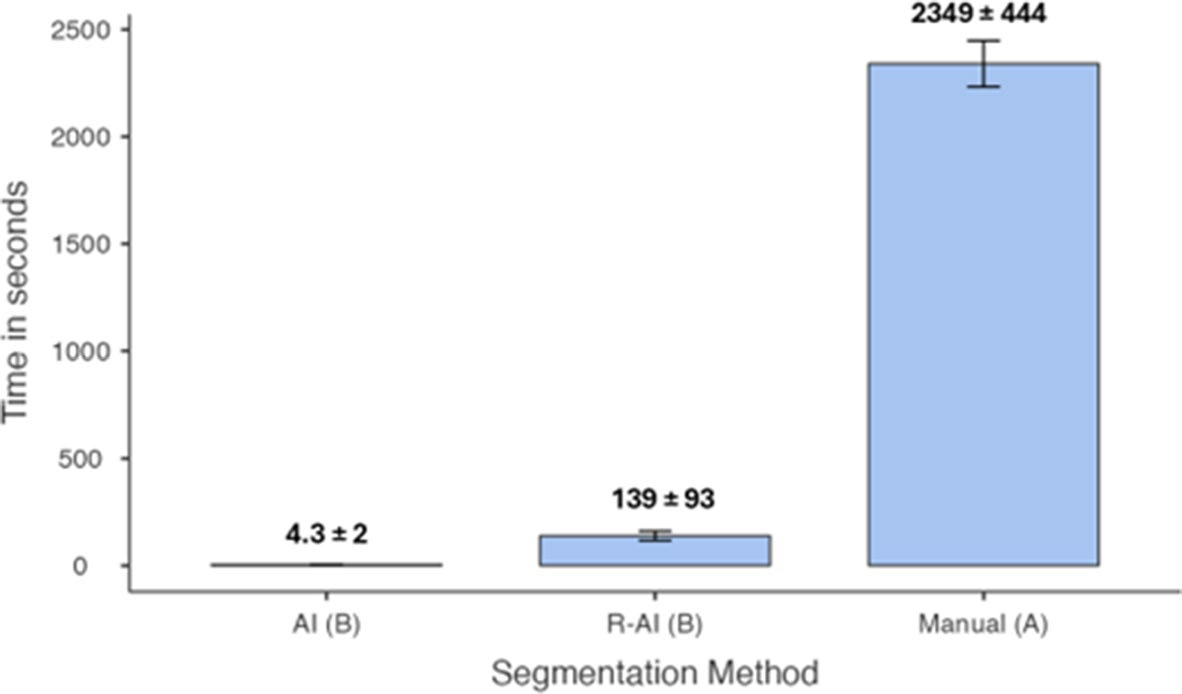
Mean ± standard deviation of time spent depending on the segmentation method. Different uppercase letters indicate a statistically significant difference among the methods (*p* ≤ .05). Abbreviations: AI, artificial intelligence; R-AI, refined artificial intelligence

Regarding the time-efficiency analysis (Fig. 6), there was a statistically significant difference between the manual segmentation and both AI-driven and R-AI segmentation methods (*p* < .05). However, no significant difference was observed between the AI-driven and R-AI segmentation methods (*p* > .05). AI-driven segmentation took 4.3 ± 2 s, while R-AI segmentation lasted 139 ± 93 s, and manual segmentation took 2349 ± 444 s.

## Discussion

Automatic segmentation of the pulp cavity system in mandibular molars presents unique challenges due to the complex anatomy and variable morphology of these teeth [22]. Our study examined the performance of an AI-driven tool designed to generate accurate 3D models of pulp cavity system in mandibular molars using CBCT scans. The results achieved demonstrate a high accuracy and efficiency, indicating that the AI-driven approach can significantly improve the endodontic workflow.

The performance of the AI-driven tool in creating 3D models of mandibular molars’ pulp cavity system was evaluated using accuracy metrics, demonstrating outstanding performance. The IoU values of 80% ± 12 for the mandibular first molars and 82% ± 10 for the mandibular second molars suggest that the tool effectively overlaps with the expert-based refined areas. The DSC of 88% ± 7 for the mandibular first molars and 90% ± 6 for the mandibular second molars reflect a high degree of agreement between the AI-generated models and R-AI models. Recall for the mandibular first molars was 86% ± 9 and for the mandibular second molars, it was 88% ± 7. Precision was equally high, with 91% ± 6 for the mandibular first molars and 93% ± 4 for the mandibular second molars. This indicates that the AI tool can accurately detect and segment the pulp cavity system with minimal inclusion of false positive and false negative voxels in the segmentation map. The overall accuracy was outstanding, with a measurement of 99% ± 1% for both types of mandibular molars. This high level of accuracy provides a strong foundation for the use of AI in clinical endodontic applications, as it can enhance the precision of treatment planning and execution. Additionally, the 95% HD showed low values of 0.14 ± 0.1 mm for the mandibular first molars and 0.15 ± 0.09 mm for the mandibular second molars, indicating a high level of precision in the 3D models.

Furthermore, the performance metrics did not differ significantly based on mandibular molar group, indicating the AI tool’s consistent accuracy across various mandibular molars. The anatomy of the pulp in the second molar tends to be more variable compared to the first molar [22]. The fact that the AI-driven tool performed equally well on both first and second molars, despite this expected variability, is a promising outcome. This result suggests that the AI model’s segmentation capabilities are robust enough to handle a range of anatomical complexities. This robustness is crucial for clinical practice, where endodontists need reliable tools that can accommodate the diverse anatomy seen in different types of mandibular molars.

The AI-driven segmentation tool demonstrated high accuracy in generating 3D pulp cavity system models of mandibular molars. Among the samples analysed, 11 of the segmented mandibular molars required no refinement, indicating that the AI-based approach accurately mapped the pulp cavity system in these cases without the need for further manual adjustments. This observation suggests that the tool effectively captured the complex anatomy of mandibular molars with minimal errors, contributing to its efficiency in endodontic planning. Additionally, for the remaining cases that did require refinement, the adjustments were minor, further highlighting the precision of the AI tool. This precision is supported by the high values of accuracy metrics, such as the DSC of 88% for mandibular first molars and 90% for mandibular second molars, and the low 95% HD measurements of 0.14 mm and 0.15 mm for mandibular first and second molars, respectively. These results confirm the tool’s capability to achieve detailed and reliable segmentation, essential for precise endodontic treatment planning.

The analysis of the required segmentation time revealed a significant advantage for the AI-driven approach in terms of time-efficiency. The AI-driven tool took only 4.3 ± 2 s to segment mandibular molars, demonstrating remarkable efficiency in generating 3D pulp cavity system models. Additionally, the R-AI method, which involves some level of refinement or verification after the initial AI-based segmentation, required 139 ± 93 s, while the manual method lasted 2349 ± 444 s. Despite its rapid performance, the AI-driven approach maintains a high level of accuracy, as evidenced by our results. The high IoU and DSC values, combined with low 95% HD values, suggest that the AI-driven tool’s speed does not compromise accuracy.

Only a limited number of studies have examined the effectiveness of AI-based tools for automatic segmentation of the pulp cavity system on CBCT scans. Duan et al. (2021) demonstrated satisfactory performance of a CNN model in segmenting teeth and pulp for single and multirooted teeth. However, their dataset was derived from a single CBCT device with similar acquisition parameters [17]. By contrast, this study used a variety of acquisition parameters, allowing the training and testing of AI-driven tool on a more diverse dataset. This broader range of imaging conditions promotes greater generalizability and reduces the risk of overfitting, resulting in models that can adapt to different clinical scenarios and equipment configurations. The use of multiple acquisition parameters enhances the reliability and robustness of our AI-based tools for segmentation of the pulp cavity system on CBCT scans. However, it should be noted that our dataset was sourced from specific CBCT devices, which might limit the generalizability of our results to other CBCT systems. Another limitation of this study was the relatively small training dataset used for the AI model. The time-consuming nature of manually segmenting the pulp cavity in multi-rooted teeth likely contributed to this constraint. A larger, more diverse dataset could help improve the generalizability and performance of the model, thereby increasing the robustness of the AI-driven tool in a clinical scenario.

Furthermore, Fontenele et al. (2022) demonstrated that the presence of fillings significantly influenced tooth segmentation performance. This finding highlights the importance of considering factors such as dental fillings when developing and validating AI-driven segmentation tools for dental imaging [11]. Our study excluded CBCT scans with high artefact expression, indicating the need for further research to evaluate the AI tool’s performance in a scenario with high expression of artefacts due to the presence of high-density and high atomic number materials. Such investigations are crucial for ensuring the broader applicability and robustness of AI-driven segmentation tools across various clinical scenarios. By addressing the impact of fillings on segmentation performance, AI-driven tools can offer more accurate and dependable support to clinicians in treatment planning and execution, ultimately leading to improved patient care outcomes.

Moreover, in terms of segmentation accuracy, the comparison with manual segmentation accuracy, a traditional segmentation method, provides additional context for assessing the performance of the AI-driven tool. Previous studies have utilized manual segmentation as a reference standard due to its human oversight and meticulous refinement. Fontenele et al. (2023) reported excellent accuracy metrics for automated segmentation of maxillary alveolar bone, despite being slightly inferior to manual segmentation, although still showing significant time efficiency with the AI-driven method being 116 times faster [13]. Additionally, Shujaat et al. (2021) showed that the CNN model exhibited optimal precision and recall for segmentation of pharyngeal airway space, with a maximal difference between automatic segmentation and ground truth of 0.98 ± 0.74 mm for HD [23]. Our study showed excellent results for the AI segmentation method, with a 546-fold and 17-fold decrease in segmentation time compared to manual and R-AI methods, respectively, while still maintaining excellent accuracy metrics.

## Conclusion

The AI-driven tool for automatically segmenting the pulp cavity system of mandibular molars demonstrated high accuracy and time efficiency, suggesting its potential to enhance the endodontic workflow. With a continuous learning process, this technology could become a valuable addition to endodontic practice, providing clinicians with a reliable and efficient method for segmenting complex pulp cavity systems. While these initial findings are encouraging, more research is needed to fully assess its impact on minimally invasive procedures, clinical outcomes, and communication between professionals and patients.

## Data Availability

Data of the current study is available upon reasonable request to the corresponding author.
